# An Account of Diseases in an Eastern District of London, from June 20, to July 20, 1808

**Published:** 1808-09

**Authors:** 


### *An Account of Diseases in an Eastern District of London, from June 20, to July 20, 1808.*

| | |
|---|---|
| ACUTE DISEASES. | |
| Ephemera - - | 3 |
| Tetanus - - | 1 |
| Hæmoptysis - - | 3 |
| Ophthalmia - - | 4 |
| Rheumatismus Acutus | 5 |
| CHRONIC DISEASES. | |
| Tussis - - - | 10 |
| Tussis cum Dyspnœa | 8 |
| Phthisis Pulmonalis | 4 |
| Gastrodynia | |

| | | | |
|---|---|---|---|
| Gastrodynia - - | 7 | PUERPERAL DISEASES. | |
| Enterodynia - - | 9 | Menorrhagia Lochialis | 5 |
| Diarrhœa - - | 17 | Ephemera - - | 3 |
| Vertigo - - - | 2 | Diarrhœa - - | 5 |
| Hemiplegia - - | 3 | Hæmorrhois - - | 2 |
| Dysuria - - - | 7 | INFANTILE DISEASES. | |
| Fluor Albus - - | 9 | Febres Mesenterica - | 2 |
| Menorrhagia - | 5 | Ophthalmia - - | 4 |
| Amenorrhœa - - | 4 | Hernia - - - | 1 |
| Herpes - - - | 3 | Convulsio - - | 2 |
| Rheumatismus Chronicus | 9 | | |

The case of Tetanus referred to in the list, was of that species commonly denominated Opisthotonos. The subject of it was a young man engaged in a mechanical employment. This disease, though it occurs most frequently in warm climates, yet is occasionally to be met with in all situations. It has been observed to appear with particular frequency, during any sudden viscissitudes in the temperature of the air, and has not unfrequently been traced to the sudden application of cold to a body greatly heated by exercise. In our own country, it may often be attributed to the injury of a nerve, by puncture or laceration.

It generally approaches with a sense of stiffness in the back of the head and neck; which, by degrees, extends to the neighbouring muscles: the head is drawn backwards, a rigidity of the muscles of the lower jaw succeeds, and by degrees the spasm becomes so strong, as to close the teeth, and produce what is called the locked jaw. In the instance referred to, (as before observed) the disease assumed all the characters of that species, which is termed opisthotonos. Besides the affection of the muscles of the neck, already mentioned, those along the spine became affected also, and the trunk of the body was very strongly drawn backwards.

Every attempt to remove the disease by anodyne and antispasmodic remedies prove fruitless, and the patient fell a victim to the disease. To the question which was repeatedly put to him, whether he had received any slight wound or injury, he replied in the negative; but upon lifting him into a warm bath, a mark was discovered in one of his feet, which he said was occasioned by a nail running into it some time before. This circumstance afforded a sufficient explanation of the cause of the symptoms which have been related.

